# Long-term treatment response after intravitreal bevacizumab injections for patients with central serous chorioretinopathy

**DOI:** 10.1371/journal.pone.0238725

**Published:** 2020-09-08

**Authors:** Hae Min Kang, Jeong Hoon Choi, Hyoung Jun Koh, Sung Chul Lee

**Affiliations:** 1 Department of Ophthalmology, Catholic Kwandong University College of Medicine, International St. Mary’s Hospital, Incheon, Republic of Korea; 2 Choikang Seoul Eye Clinic, Seoul, Republic of Korea; 3 Department of Ophthalmology, Yonsei University College of Medicine, Seoul, Republic of Korea; 4 Department of Ophthalmology, Konyang University College of Medicine, Daejeon, Republic of Korea; Massachusetts Eye & Ear Infirmary, Harvard Medical School, UNITED STATES

## Abstract

**Purpose:**

To investigate long-term treatment response after intravitreal bevacizumab injections (IVBIs) for central serous chorioretinopathy (CSC).

**Methods:**

This retrospective, interventional study investigated the medical records of 45 eyes of 44 patients with CSC who underwent IBVIs and completed at least 2-year follow-up period. Complete resolution (CR) was defined as complete resolution of subretinal fluid at least 3 months after the last IVBI. Thick-choroid CSC was defined as mean subfoveal choroidal thickness more than 300.0 *μ*m. The main outcome measure was long-term treatment outcome after IVBIs in patients with CSC.

**Results:**

Thirty-five patients (79.5%) were male, and their mean age was 45.5 ± 9.6 years. The mean follow-up period was 35.1 ± 11.5 months. Twenty-two eyes (48.9%) had acute CSC, and 40 eyes (88.9%) achieved CR. Twenty eyes (50.0%) developed recurrence, the mean number of IVBIs to achieve the first CR was not significantly different between eyes with and without recurrences (2.6 ± 1.6 vs. 2.9 ± 1.9; P = 0.658). Thick-choroid CSC was significantly difference between the eyes with and without recurrence (17 eyes, 85.0% vs. eyes, 50.0%; P = 0.020). Among the baseline characteristics, serous pigment epithelial detachment (B = - 2.580, P = 0.032) and thick-choroid (B = 1.980, P = 0.019) were significantly associated with recurrence.

**Conclusion:**

Eyes with CSC treated with IVBI and achieving complete resolution of subretinal fluid have 50% chance of recurrence in the long term. Thinner choroid and serous pigment epithelial detachment appear protective for recurrences.

## Introduction

Central serous chorioretinopathy (CSC) is characterized by serous neurosensory retinal detachment at the posterior pole due to leakage from the retinal pigment epithelium (RPE) [1–5]. Serous neurosensory macular detachment in patients with CSC leads to visual symptoms involving metamorphopsia, blurred vision, and micropsia in a relatively young and middle-aged population [1–5]. The majority of cases of acute CSC resolve spontaneously within 3 months [6–8]; however, early intervention for acute CSC has been advocated to prevent irreversible visual loss [9, 10]. About 10% of patients with acute CSC have multiple recurrences or progression to a chronic course [4, 11]. Persistent serous retinal detachment in a patient with chronic CSC can lead to permanent visual impairment due to RPE decompensation and disruption of the photoreceptor ellipsoid zone [1, 2, 12, 13]. Treatment modalities for CSC include focal laser photocoagulation, photodynamic therapy (PDT), and intravitreal anti-vascular endothelial growth factor (VEGF) injections [14].

Although some have evaluated the treatment efficacy of intravitreal anti-VEGF injections for CSC [15–23], clinical outcome of anti-VEGF therapy for CSC have been inconsistent. Some studies have indicated that intravitreal bevacizumab injections (IVBIs) may be helpful for rapid resolution of sensory retinal detachment resulting in visual recovery, as 42–82.5% of a study population showed complete resolution of subretinal fluid (SRF) [15–23]. However, other studies have not shown a beneficial effect of IVBIs in patients with CSC [24–28]. One study found no significant difference at the 6- and 12-month follow-up visit between IVBIs and observation [26]. Other studies demonstrated a superior visual outcome in patients subjected to observation compared to those who received IVBIs [27, 28]. Other anti-VEGF agents, such as ranibizumab and aflibercept, have also been used for chronic CSC and seem to be more effective for treatment-naïve or non-responsive cases than IVBIs with a short follow-up period [29–31]. In addition, a few randomized studies have shown no superior efficacy of anti-VEGF injections for CSC [28, 32].

In contrast to the mixed, varied clinical efficacy of intravitreal anti-VEGF injections, PDT shows superior and consistent treatment outcomes [33–35]. Thus, PDT is considered a good treatment option for patients with CSC. However, PDT requires specialized equipment, such as a PDT laser machine, and possible complications include chorioretinal atrophy, secondary choroidal neovascularization, and choroidal hypoperfusion [36–39]. Thus, intravitreal anti-VEGF agents cannot be excluded as a treatment option for patients with CSC.

Although several studies have investigated the clinical efficacy of intravitreal anti-VEGF agents for patients with CSC, no study has investigated the long-term treatment outcomes of these patients. In this study, we investigated the long-term treatment outcome of IVBIs for patients with CSC. In addition, we investigated the rates of recurrence and predictive factors for recurrence in these patients.

## Methods

### Enrollment of study subjects

This retrospective interventional study was performed at the Catholic Kwandong University College of Medicine, International St. Mary’s Hospital. We retrospectively reviewed the medical records of patients with macular-involved CSC who underwent IVBIs and completed at least a 2-year follow-up. An IVBI was performed at 4-week intervals until complete resolution of SRF. Then, IVBIs were performed on an as-needed basis as determined using spectral domain optical coherence tomography (SD OCT) findings, such as the reappearance of SRF. The exclusion criteria were: 1) other concomitant eye diseases, such as age-related macular degeneration, polypoidal choroidal vasculopathy, diabetic retinopathy, pathological myopia, or retinal vein occlusion, which might affect choroidal thickness; 2) no prior history of PDT; 3) the presence of systemic diseases that require long-term systemic steroid treatment; and 4) any intraocular surgery within 3 months. This study was approved by the Institutional Review Board of International St. Mary’s Hospital, Catholic Kwandong University College of Medicine, and adhered to the tenets of the Declaration of Helsinki. Because of the retrospective nature of this study, the Institutional Review Board waived the need for informed consent.

CSC is defined by choroidal congestion and choroidal hyperpermeability, which leads to focal RPE defects and the formation of serous pigment epithelium detachment. Chronic CSC is defined as a symptom duration and/or persistent serous retinal detachment of more than 3 months. Recurrence of CSC is defined as the reappearance of SRF after at least a 3-month period with complete resolution of a previous CSC. Complete resolution of CSC is defined as complete resolution of serous macular detachment or resolution of SRF by at least 3 months after the last IVBI.

We also classified the study population into two groups according to the baseline subfoveal choroidal thickness (SFCT) measured by SD OCT: Thick-choroid CSC was defined as a mean SFCT of more than 300.0 *μ*m at the time of the initial diagnosis and during the follow-up period.

The primary outcome measure was the treatment response to IVBIs during a follow-up period of at least 2 years. The secondary outcome was the appearance following IVBIs of possible factors correlated with the recurrence of CSC.

### Ocular examination

An ophthalmologic examination, including a slit-lamp examination, an intraocular pressure measurement using a non-contact tonometer, and a fundus examination after pupillary dilation were performed at each visit. The refractive error of each eye was measured using an autorefractor and converted to spherical equivalents [diopters (D)]. Fundus autofluorescence images and SD OCT (Spectralis; Heidelberg Engineering, Heidelberg, Germany) were taken with an enhanced depth imaging modality. Both fluorescein angiography and indocyanine green angiography were performed using the Heidelberg Retina Angiograph system (HRA-2; Heidelberg Engineering) with a confocal scanning laser ophthalmoscope for diagnosis of CSC at the first visit. At each follow-up visit, the best-corrected visual acuity (BCVA) of the affected eye(s) with CSC was checked with a decimal visual acuity chart, and then each value was converted to a logarithm of the minimum angle of resolution (logMAR).

### Evaluation by spectral domain optical coherence tomography

Serial cross-sectional horizontal scans of approximately 121 *μ*m apart in a 30 × 30° macular area were obtained for the macular evaluation. Single horizontal and vertical scans across the fovea were also obtained separately.

At least two good-quality horizontal and vertical scans across the fovea were obtained for each eye to measure the SFCT. The choroidal thickness was defined as the perpendicular distance from the outer border of the hyper-reflective line, corresponding to the RPE, to the chorio-scleral interface. Using digital calipers provided by the Heidelberg Spectralis OCT software, the choroidal thickness was measured horizontally and vertically at the subfoveal region in each trans-sectional image, and the average measurement was calculated. Two independent observers (HMK and JHC), who were blinded to the clinical data, measured the choroidal thickness.

The central macular thickness (CMT) was defined as the mean retinal thickness in the central subfield, a region with a diameter of 1.0 mm around the fovea. The inner and the outer rings had diameters of 3.0 mm and 6.0 mm, respectively. The CMT in the 9-standard early treatment diabetic retinopathy study (ETDRS) macular grid subfields was automatically calculated by the Heidelberg software. All SD OCT images were reviewed for any possibility of segmentation errors, and the ETDRS grid was manually centered at the fovea.

The height of the SRF, presence of flat irregular pigment epithelial detachment (PED), presence of serous PED, presence of hyper-reflective speckles in the SRF, and presence of RPE humps were evaluated in each eye. The height of the SRF was manually measured at the highest point between the photoreceptor ellipsoid zone and the RPE. The presence of choroidal hyperpermeability was evaluated by ICGA. Choroidal hyperpermeability was defined as focal areas of hyper-fluorescence with blurred margins on mid- and late-phase images. Classic CSC was defined as focal or multifocal detachment of the neurosensory retina on fluorescein angiography (FA), or an RPE with single or multifocal areas of leakage at the late phase.

### Statistical analysis

The data are presented as mean ± standard deviation (range). Baseline characteristics included age, sex, refractive error, BCVA, and chronicity of CSC at the time of the initial visit. The mean SFCT, the greatest height of the SRF, and various imaging characteristics, such as choroidal hyperpermeability, flat irregular PED, and serous PED, were also evaluated. IBM SPSS Statistics software for Windows, version 22.0 (IBM Corp., Somers, NY, USA) was used for statistical analyses. The Kruskal–Wallis test was used for continuous variables, and the chi-square test was used for categorical variables when the clinical characteristics were compared between eyes with and without recurrence. Repeated-measured analysis of variance was used to compare the mean BCVA, the mean SFCT, and the mean CMT during the follow-up period. To investigate the possible predictive factors for the recurrence of CSC after the IVBIs, binary regression analysis using the forward analysis method was used. Mauchly's test of sphericity and Kolmogorov–Smirnov analyses were used to confirm statistical validity. A P-value < 0.05 was considered significant.

## Results

### Baseline characteristics of the study population

A total of 45 eyes from 44 patients with CSC were included in this study. The mean age at the time of diagnosis was 45.5 ± 9.6 years (20–59 years), and the mean follow-up duration was 35.1 ± 11.5 months (24–68 months). Twenty-two eyes (48.9%) had acute CSC, and 40 eyes (88.9%) achieved complete resolution. The mean BCVA was 0.2 ± 0.2 (0–0.6) logMAR at baseline, and 0.1 ± 0.1 (0–0.4) logMAR at 24 months (P < 0.001) after a mean of 4.4 ± 3.4 (1–17) IVBIs in overall population. None of the study population showed choroidal neovascularization (CNV).

In 40 eyes with at least one complete resolution, the mean number of IVBIs to the first complete remission was 2.7 ± 1.7 injections (1–7 injections). The mean BCVA was 0.2 ± 0.2 (0–0.6) logMAR at baseline, and 0.1 ± 0.2 (-0.1–0.6) logMAR at final visit (P = 0.018). The mean CMT was 387.6 ± 105.0 (198.0–661.0) ㎛ at baseline, and 252.2 ± 47.1 (178.0–410.0) at final visit (P < 0.001). The mean SFCT was 402.7 ± 127.5 (243.0–646.0) ㎛ at baseline, and 377.5 ± 129.7 (247.0 to 641.5) ㎛ at final visit (P < 0.001).

Five patients (11.1%) did not achieve complete resolution of CSC after the IVBIs, and were referred for PDT. The detailed clinical characteristics of these five patients are shown in Table 1.

**Table 1:** Characteristics of the five patients who did not have at least one complete resolution of central serous chorioretinopathy after the intravitreal bevacizumab injections.

	Patient 1	Patient 2	Patient 3	Patient 4	Patient 5
Age (years)	53	39	41	42	60
Sex	Male	Female	Female	Male	Male
Chronicity	Acute	Acute	Chronic, recurrent	Chronic, recurrent	Chronic recurrent
Previous treatment			Observation	Observation	Focal laser
Refractive errors (diopters)	0	–0.25	–0.25	–3.75	1.25
Total number of IVBIs	10	11	11	12	10
BCVA at the time of diagnosis (logMAR)	0.3	0.2	0.2	0.3	0.2
BCVA at last visit (logMAR)	0.3	0.1	0.2	0.1	0.3
SRF height at the time of diagnosis (*μ*m)	94.0	58.0	35.0	221.0	351.0
SRF height at the last visit (*μ*m)	35.0	48.0	38.0	53.0	73.0
CMT at the time of diagnosis (*μ*m)	256	380	301	443	384
Serous PED	-	-	-	-	-
Flat irregular PED	+	-	-	+	+
Choroidal hyperpermeability on ICGA	+	-	-	+	+
Hyper-reflective speckles in SRF	-	-	-	+	+
Mean SFCT at the time of diagnosis (*μ*m)	565.0	93.0	481.0	469.0	269.0
Mean SFCT at last visit (*μ*m)	525.0	71.0	422.0	417.0	238.5

Abbreviations: BCVA, best-corrected visual acuity; CMT, central macular thickness; ICGA, indocyanine green angiography; IVBI, intravitreal bevacizumab injection; logMAR, logarithm of the minimum angle of resolution; PED, pigment epithelial detachment; SFCT, subfoveal choroidal thickness; SRF, subretinal fluid.

### Recurrence after intravitreal bevacizumab injections for central serous chorioretinopathy

Among 40 eyes showing at least one complete resolution, 20 eyes (50.0%) developed at least one recurrence (mean 1.5 ± 0.8 recurrences; 1–2 times). The mean number of IVBIs for the first resolution was 2.9 ± 1.9 times (1–7 times) in eyes without recurrence and 2.6 ± 1.6 times (1–6 times) in those with recurrence (P = 0.658). However, the total number of IVBIs was significantly different between eyes with and without recurrence (mean 5.2 ± 3.1 vs. 2.9 ± 1.9 times, respectively; P = 0.010). The mean duration between the first complete resolution to the first recurrence was 11.6 ± 6.4 months (4–43 months). The mean BCVA at baseline was 0.2 ± 0.2 (0–0.6) logMAR in the eyes with recurrence, and 0.2 ± 0.2 (-0.1–0.4) logMAR in the eyes without recurrence (P = 0.620). The mean BCVA at the final visit was 0.1 ± 0.2 (0–0.6) logMAR in the eyes with recurrence, and 0.1 ± 0.2 (-0.1–0.4) in the eyes without recurrence (P = 0.495).

The mean height of the SRF at baseline was 189.7 ± 111.0 *μ*m (29.0–487.0 *μ*m) in eyes without recurrence, and 150.8 ± 101.3 *μ*m (32.0–378.0 *μ*m) in eyes with recurrence (P = 0.224). The mean CMT was not significantly different between eyes with and without recurrence at baseline (379.5 ± 109.0 vs. 406.9 ± 100.5 *μ*m, respectively; P = 0.165) and at the final visit (264.9 ± 54.7 vs. 238.8 ± 34.1 *μ*m, respectively; P = 0.194). The mean SFCT was significantly thicker in eyes with recurrence than in those without recurrence at baseline (441.9 ± 96.9 vs. 366.8 ± 144.1 *μ*m, respectively; P = 0.043) and the final visit (416.2 ± 108.5 vs. 332.7 ± 139.0 *μ*m, respectively; P = 0.028).

### Comparison of baseline characteristics between eyes with and without recurrence of central serous chorioretinopathy after intravitreal bevacizumab injections

After assessing the recurrence rate and comparing the clinical outcomes between the eyes with and without recurrence, we compared the baseline characteristics in the eyes with and without recurrence after IVBIs for CSC.

No significant differences in baseline clinical characteristics were observed, including the mean age at the time of diagnosis, sex, total follow-up period, and mean symptom duration (P = 0.212, P = 0.531, P = 0.870, and P = 0.273, respectively). Twelve eyes (60.0%) with acute CSC did not develop a recurrence, and six eyes (30.0%) with acute CSC developed a recurrence (P = 0.055).

We also compared the ocular imaging characteristics between the eyes with and without recurrence in these patients. Serous PED was present in six (30.0%) of the eyes without recurrence, and in one (5.0%) of the eyes with a recurrence (P = 0.046). We also classified the eyes into relatively thin-choroid CSC (mean SFCT < 300.0 *μ*m) and thick-choroid CSC (mean SFCT ≥ 300.0 *μ*m), and the presence of thick-choroid CSC was higher in eyes with recurrence than in those without: 13 eyes (85.0%) vs. 10 eyes (50.0%), respectively (P = 0.020). A detailed comparison of the baseline characteristics of eyes with and without a recurrence is shown in Table 2.

**Table 2:** Comparison of baseline characteristics between eyes with and without recurrence after complete remission with intravitreal bevacizumab injections (IVBIs) for central serous chorioretinopathy.

	Eyes without recurrence (20 eyes)	Eyes with recurrence (20 eyes)	P-value
Mean age at the time of diagnosis (years)	47.2 ± 10.1	43.3 ± 9.1	0.212*
Mean refractive error (diopters)	−0.7±1.6	−0.4 ± 1.2	0.562*
Mean symptom duration (months)	2.3 ± 3.1	5.5 ± 11.5	0.273*
Mean number of IVBIs to the first remission	2.9 ± 1.9	2.6 ± 1.6	0.658*
Mean total number of IVBIs	2.9 ± 1.9	5.2 ± 3.1	0.010*
Acute CSC (%)	12 (60.0%)	6 (30.0%)	0.055†
Hyper-reflective speckles in the SRF (%)	9 (45.0%)	6 (30.0%)	0.257†
Choroidal hyperpermeability on ICGA (%)	5 (30.0%)	9 (45.0%)	0.160†
Flat irregular PED (%)	3 (15.0%)	6 (30.0%)	0.225†
Serous PED (%)	6 (30.0%)	1 (5.0%)	0.046†
RPE humps (%)	13 (65.0%)	8 (40.0%)	0.102†
Thick choroid (%)	10 (50.0%)	17 (85.0%)	0.020†

Abbreviations: CSC, central serous chorioretinopathy; ICGA, indocyanine green angiography; PED, pigment epithelial detachment; RPE, retinal pigment epithelium; SRF, subretinal fluid.

*The Mann–Whitney *U-*test for continuous variables, and the †chi-square test for categorical variables were used for the statistical analysis. A P-value < 0.05 was considered significant.

### Factors associated with recurrence after the intravitreal bevacizumab injections for central serous chorioretinopathy

We investigated the baseline factors that were significantly associated with recurrence after IVBIs for CSC. Among the various baseline characteristics, the presence of serous PED (B = -2.580, P = 0.032) and thick-choroid CSC (B = 1.980, P = 0.019) were significantly associated with recurrence after IVBIs in these patients (Table 3).

**Table 3:** Possible baseline predictive factors for recurrence after intravitreal bevacizumab injections for patients with central serous chorioretinopathy during a long-term follow-up period by binary logistic regression analysis.

Factors	β	P-value
Chronicity	1.029	0.310
BCVA at baseline	0.315	0.574
Height of SRF at baseline	0.152	0.697
CMT at baseline	0.064	0.800
Hyper-fluorescent speckles in SRF	3.496	0.062
Choroidal hyperpermeability on ICGA	0.002	0.969
Mixed autofluorescence in FAF images	0.427	0.513
Flat irregular PED	0.093	0.760
Serous PED	-2.580	0.019
RPE humps	1.448	0.229
Classic FA patterns	2.051	0.152
Mean SFCT at baseline	1.560	0.212
Thick choroid	1.980	0.019

Abbreviations: BCVA, best-corrected visual acuity; CMT, central macular thickness; FA, fluorescein angiography; ICGA, indocyanine green angiography; logMAR, logarithm of the minimum angle of resolution; PED, pigment epithelial detachment; RPE, retinal pigment epithelium; SFCT, subfoveal choroidal thickness; SRF, subretinal fluid.

**Fig 1:**
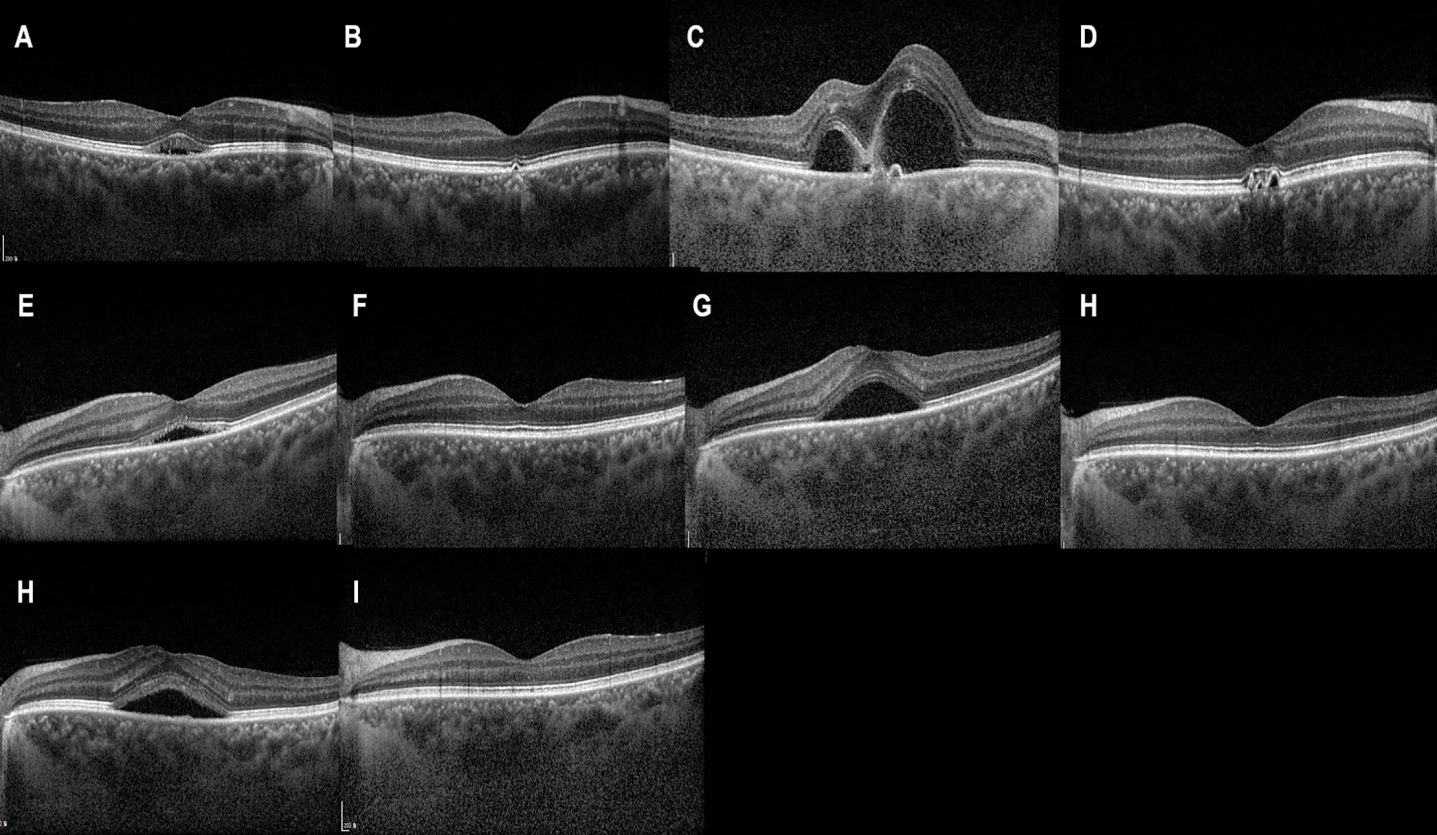
Image from 37-year-old male patient with central serous chorioretinopathy (CSC) in both eyes. The patient had a history of CSC 11 years prior that resolved spontaneously. He also had a recent recurrence of CSC in both eyes for 4 months and was referred to our hospital due to persistent subretinal fluid (SRF) in the right eye. The best-corrected visual acuity (BCVA) was 0.1 logarithm of the minimum angle of resolution (logMAR) in the right eye. (A) Subretinal fluid (SRF) at fovea with photoreceptor ellipsoid zone blurring was noted. Hyper-reflective dots in SRF was also noted. (B) A single intravitreal bevacizumab injection (IVBI) led to complete resolution of SRF, but small retinal pigment epithelial detachment (PED) was noted. (C) After 11 months, CSC recurred showing profuse subretinal fluid, and two more IVBIs were performed to resolve the SRF (D). (E) SRF also developed in the left eye, and the BCVA was 0.1 logMAR at the time of recurrence. After the first IVBI, SRF resolved completely (F), however, SRF recurred after 1 month (G). (H) Two more IVBIs were needed in the left eye to resolve the SRF, and (I) CSC recurred after 18 months. (J) Two IVBIs were needed to resolve the SRF of the recurred CSC.

**Fig 2:**
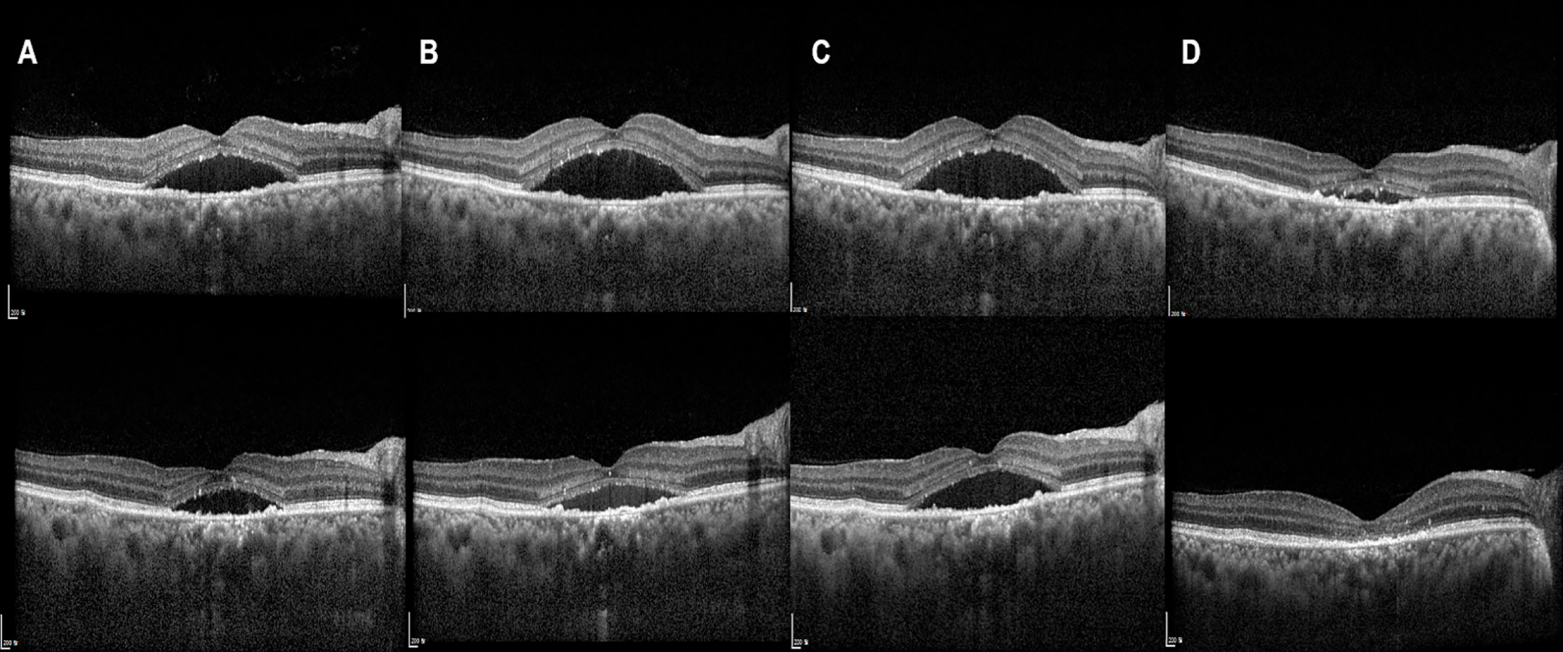
Image from a 49-year-old male patient with central serous chorioretinopathy (CSC) in the right eye. The subretinal fluid (SRF) in the right eye did not resolve after 3 months, and the patient was referred to our hospital. At the time of diagnosis, the best-corrected visual acuity (BCVA) was 0.2 logarithm of the minimum angle of resolution (logMAR) in the right eye. (A) Subretinal fluid (SRF) with retinal pigment epithelium humps and focal photoreceptor ellipsoid zone disruption was noted. Intravitreal bevacizumab injection (IVBI) was performed, but SRF was aggravated (B). Second IVBI also did not improve SRF (C), however, 3^rd^ IVBI much improved SRF (D). Subsequent 4^th^ (E), 5^th^ (F), 6^th^ (G) IVBI did not completely resolve SRF, but 7^th^ IVBI led to complete resolution of SRF, and CSC did not recur for 15 months after last IVBI. The BCVA was 0.3 logMAR at the last visit.

**Fig 3:**
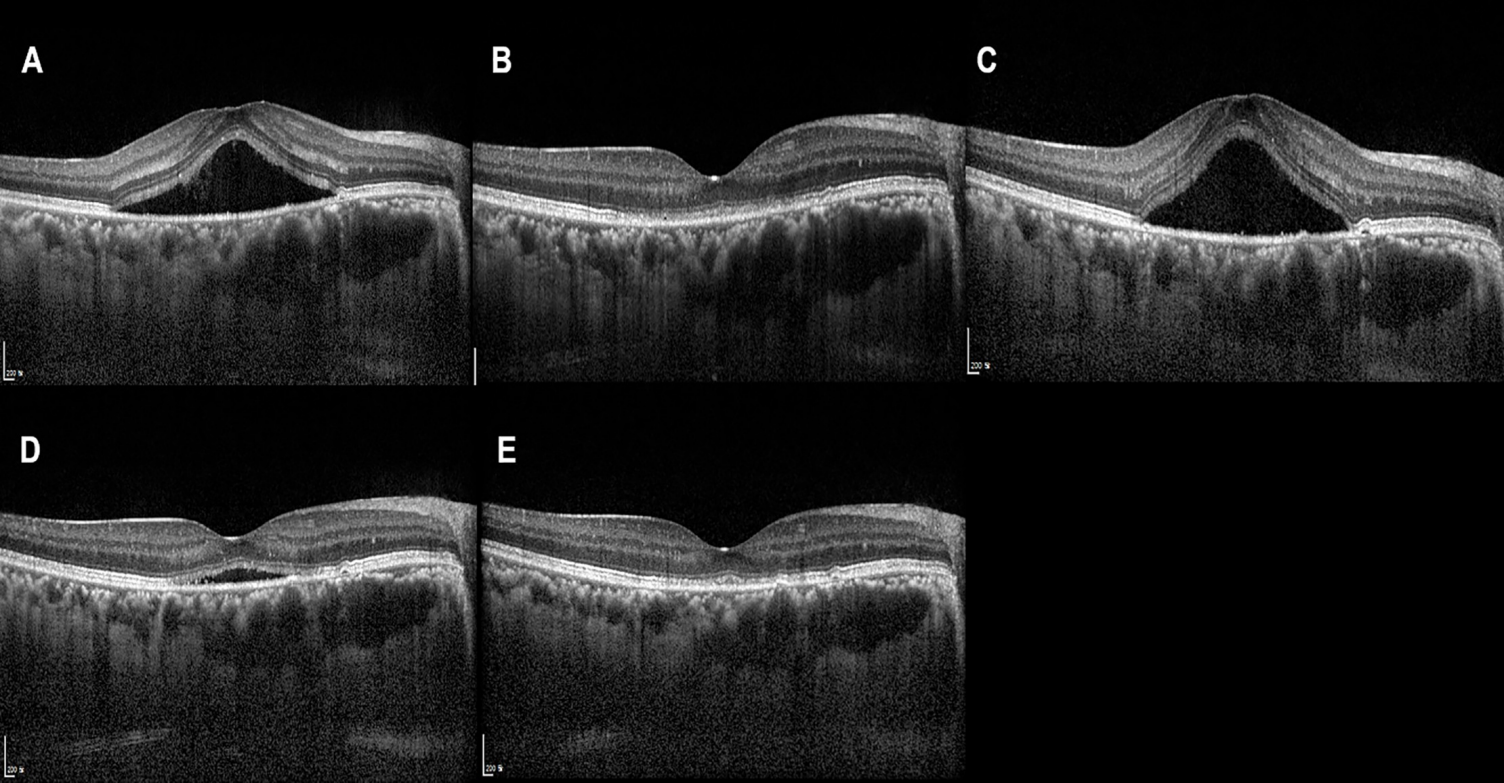
Image from a 34-year-old female patient with acute central serous chorioretinopathy (CSC) in the right eye. She complained of blurred vision in the right eye for 2 months, and the best-corrected visual acuity (BCVA) was 0.3 logarithm of the minimum angle of resolution (logMAR). (A) Subretinal fluid (SRF) with hyper-reflective dots was noted. (B) A single intravitreal bevacizumab injection (IVBI) led to resolution of SRF, and the BCVA improved to 0 logMAR. (C) After 24 months, CSC recurred, and the BCVA in the right eye was 0.5 logMAR. (D) IVBI was performed, but SRF did not completely resolve, and (E) additional IVBI was needed to resolve the SRF, and the BCVA improved to 0.2 logMAR.

Representative figures are shown in Figs 1–3.

## Discussion

In this study, we evaluated the long-term treatment response to IVBIs in patients with CSC, and investigated the baseline characteristics predictive of recurrence in this study population. During a follow-up period of at least 2 years, 88.9% of the patients with CSC developed at least one complete resolutionwith a mean of 2.7 IVBIs. During the mean follow-up period of 35 months, half of the eyes with at least one complete resolution showed at least one recurrence. The final BCVA was not significantly different between eyes with and without recurrence, although the eyes without recurrence showed better visual outcomes. The mean SFCTs at baseline and the final visit were significantly thicker in eyes with a recurrence than in those without. Among the various baseline factors, the presence of serous PED and the thin-choroid type of CSC was negatively correlated with recurrence of CSC.

The long-term treatment results of this study suggest that IVBIs can remain as a treatment option for patients with CSC. Previously, some studies have investigated the cytokines in the aqueous humor of patients with CSC, resulting in inconsistent results [40–43]. Some studies suggest that aqueous VEGF level was not significantly elevated in the patients with CSC, supporting ineffectiveness of anti-VEGF therapy in these patients [40, 41]. However, other studies have shown different findings. One study demonstrated that the VEGF-A level is significantly higher in bevacizumab responders than in non-responders, suggesting that VEGF may contribute to the pathogenesis of CSC [42]. Another study found that proinflammatory cytokines, such as interleukin (IL)-6 and IL-8, are significantly upregulated in patients with chronic CSC compared to those with acute CSC [43]. Angiogenic cytokines and VEGF-A are also upregulated more in patients with CSC than in those with acute CSC [43]. IVBIs significantly reduce choriocapillaris endothelial cell fenestrations [44, 45]. In addition, SFCT, choroidal vascular hyperpermeability, and choroidal blood flow decrease significantly after IVBIs in patients with CSC [17, 22, 46–49]. Along with these previous studies, majority of our study population showed at least one complete resolution, with a maximum of seven IVBIs. Repeated IVBI treatments in some patients in our study also led to a final complete resolution of CSC.

In addition to the clinical efficacy of IVBIs, the possible predictive factors for patients being good responders to IVBIs have been investigated in patients with CSC. These predictive factors, such as hyperfluorescence on ICGA, classic FA findings, and a relatively thick SFCT, have been reported in multiple studies [16, 17, 46, 47]. Some studies have shown that presence of CNV can be more responsive for anti-VEGF therapy [50, 51], none of our study population showed CNV. In contrast to the previously known factors for good responders, we found that the presence of serous PED and thin-choroid CSC were significantly associated with a low possibility of recurrence. however, Our study demonstrated favorable results of the IVBIs, which is inconsistent with previous studies, suggesting that a thicker SFCT is predictive of patients being good responders to IVBIs [17, 46, 47]. However, the majority of our study population was considered to be good responders to IVBIs: nearly 90% of the patients showed at least one complete resolution after the IVBIs. Thus, among these CSC patients who responded well to the IVBIs, relativelythinner baseline SFCT may reflect a weaker disease activity, resulting in less recurrence during the long-term follow-up period.

Our study is the first attempt to investigate the presence of relatively thin-choroid CSC and its clinical significance to IVBIs. The results of this study show that relatively thin-choroid CSC occurred among the study population, and the presence of thick-choroid CSC seemed to be associated with higher possibility of recurrence after IVBIs. Because the mean SFCT appears to reflect disease activity, such as choroidal hyperpermeability in patients with CSC [52], relatively thin-choroid CSC may indicate lower disease activity in these patients. Relatively thin choroid in eyes with CSC may indicate lower hydrostatic pressure. Although the exact pathophysiology of CSC is still under investigation, RPE decompensation and choroidal hyperpermeability seem to be involved [2, 53, 54]. Increased choroidal extravasated fluid from the hyperpermeable choroidal vessels induces an elevation of hydrostatic pressure in the choroid. This elevated hydrostatic pressure exerts mechanical stress on the RPE layer, leading to RPE detachment by fluid that is forced anteriorly through Bruch’s membrane. If rupture or permeability of the RPE increases, it leads to leakage into the subretinal space, resulting in serous retinal detachment [2, 53, 54]. Thus, thickening of the choroid in a patient with CSC may reflect more hydrostatic pressure associated with choroidal hyperpermeability, suggesting higher disease activity. Then, it can be speculated that patients with relatively thin-choroid CSC have less disease activity than those with thick-choroid CSC at the time of disease development, leading to a good treatment response and less of a possibility of recurrence after IVBIs in these patients. However, this cannot fully explain the treatment response of CSC to the IVBIs, because two of five eyes that did not show complete resolution had relatively thin-choroid CSC. Thus, relatively thin-choroid CSC cannot entirely explain the treatment response to IVBIs, and further studies are needed to clarify the clinical significance of relatively thin-choroid CSC. In addition, the treatment response of relatively thin-choroid CSC and thick-choroid CSC to PDT and/or intravitreal anti-VEGF injections should be further investigated.

In addition to relatively thin-choroid CSC, the presence of serous PED was negatively associated with a recurrence of CSC in our study. During the pathogenesis of CSC, serous PED can occur due to hydrostatic pressure associated with choroidal hyperpermeability [2, 53, 54]. In our study, acute CSC tended to be more prevalent in eyes without recurrence than in those with recurrence, but it did not reach statistical significance. However, serous PED tended to be more prevalent in acute CSC (N = 5, 27.8%) than chronic CSC (N = 2, 9.1%; P = 0.130). Serous PED in CSC has been observed to spontaneously regress in more than 60% of cases over a long-term follow-up period [55], and serous PED in chronic CSC may become vascularized PED [56].Thus, the presence of serous PED may indirectly reflect the chronicity of CSC. We speculate that more acute CSC may recur less long-term after IVBIs by reducing choroidal hyperpermeability in the early stage of the disease. This result also indirectly supports previous results that emphasized the importance of early intervention for CSC [9, 10].

Another interesting point is that the mean BCVA was not significantly different in eyes with and without recurrence in our study population. In the overall study population, IVBIs led to significant visual improvement during the follow-up period. The BCVA in each patient did not decrease much during the follow-up period, even in patients without complete resolution. Thus, the visual deterioration associated with CSC may not be rapid regardless of the treatment response or recurrence during continuous IVBI treatment. However, our patients underwent repeated treatment with IVBIs, and vision changes cannot be the deciding point for treatment. Persistent serous retinal detachment and the resulting disruption of the photoreceptor ellipsoid zone can lead to permanent visual deterioration in the patients with CSC. Thus, timely intensive treatment for CSC is needed to prevent permanent visual loss despite vision changes, and the decision to change the treatment regimen is important in these patients.

However, we cannot conclude that anti-VEGF therapy is superior to PDT, combination therapy of PDT with anti-VEGF agents, or observation because our study lacks the control groups. This study was not comparative, and about 10% of the study population did not show complete resolution despite repeated IVBIs. We terminated the IVBIs and referred the latter patients to other hospitals at which PDT was available. In addition, it is possible that early intervention with PDT might have shortened the treatment period in some of our patients who underwent repeated IVBIs. It is also possible that some patients with acute or chronic CSC might spontaneously resolve regardless of IVBIs. Thus, we cannot propose that IVBIs can replace PDT, combination therapy of PDT with anti-VEGF agents, or observation when treating the patients with CSC. Our study results suggest that IVBIs still can remain as a treatment option for CSC, as the treatment showed some efficacy during a long-term follow-up period. Our results suggest that IVBIs can be considered as a treatment option, even over the long-term period in the clinical settings in which PDT is not available, or for the patients who are concerned about PDT-induced complications [34–37], but request early intervention than simple observation.

This study had several limitations, including a relatively small study population and a retrospective design. Because our hospital lacks the capability to deliver PDT, we could not compare the treatment efficacies of PDT and IVBIs, which may limit the application of our findings. We also lack the control group of simple observation, because most of the our patients requested early intervention than observation. Abscence of the control group limit comparison of treatment efficacy among the treatment strategies in the patients with CSC. In addition, our study population was a mixture of patients with acute and chronic CSC. A further prospective study that compares IVBIs to PDT, combination therapy of PDT with IVBIs, and observation will be needed to elucidate the treatment efficacy in patients with CSC. In addition, intervention for acute and chronic CSC is also recommended.

In conclusion, about 90% of eyes with CSC showed at least one complete remission during a mean follow-up period of 35 months. The presence of serous PED and thin-choroid CSC at the time of diagnosis was significantly associated with a low possibility of recurrence in patients with CSC.
